# Enhancement of Phosphate Absorption by Garden Plants by Genetic Engineering: A New Tool for Phytoremediation

**DOI:** 10.1155/2013/182032

**Published:** 2013-08-04

**Authors:** Keisuke Matsui, Junichi Togami, John G. Mason, Stephen F. Chandler, Yoshikazu Tanaka

**Affiliations:** ^1^Research Institute, Suntory Global Innovation Center Limited, 1-1-1 Wakayama-dai, Shimamoto-cho, Mishima-gun, Osaka 618-8503, Japan; ^2^Safety Science Institute, Quality Assurance Division, Suntory Business Expert Limited, 57 Imaikami-cho, Nakahara-ku, Kanagawa Kawasaki 211-0067, Japan; ^3^Biosciences Research Division, Department of Environment & Primary Industries, AgriBio, Centre for AgriBioscience, 5 Ring Road, La Trobe University, Bundoora, VIC 3083, Australia; ^4^School of Applied Sciences, RMIT University, P.O. Box 71, Bundoora, VIC 3083, Australia

## Abstract

Although phosphorus is an essential factor for proper plant growth in natural environments, an excess of phosphate in water sources causes serious pollution. In this paper we describe transgenic plants which hyperaccumulate inorganic phosphate (Pi) and which may be used to reduce environmental water pollution by phytoremediation. At*PHR1*, a transcription factor for a key regulator of the Pi starvation response in *Arabidopsis thaliana*, was overexpressed in the ornamental garden plants *Torenia, Petunia, and Verbena.* The transgenic plants showed hyperaccumulation of Pi in leaves and accelerated Pi absorption rates from hydroponic solutions. Large-scale hydroponic experiments indicated that the enhanced ability to absorb Pi in transgenic torenia (At*PHR1*) was comparable to water hyacinth a plant that though is used for phytoremediation causes overgrowth problems.

## 1. Introduction

Water pollution has become a serious problem around the world. Contamination by toxic substances such as endocrine disruptors and heavy metals and excessive inflows of phosphorus, nitrogen and other elements all contribute to water pollution. Eutrophication is one of the major problems associated with water pollution and is caused by inflow of excess amounts of nutrients (especially phosphorus and nitrogen) [1]. The sources of excessive amounts of phosphorus and nitrogen are agricultural run-off, sewage, industrial effluents, and natural erosion from soil and rocks. Eutrophication is due to rapid growth of phytoplankton causing algal blooms or “red tides,” the result of which are serious environmental problems such as bad odor and fish death as a result of oxygen depletion and accumulation of toxic cyanotoxins [2]. 

Phosphorus can be removed by physical, chemical, and biological methods [3–6]. Physical and chemical methods (e.g., electrolytic, crystallization, filtration, and aggregation/separation methods) are superior in terms of removal efficiency and throughput capacity. However, these methods require complicated equipment and large quantities of chemicals, resulting in high cost and environmental burdens. A biological method, the anaerobic-anoxic-oxic method (A2O), is one of the advanced activated sludge methods and has been widely examined in sewage plants. However this method is also very expensive [7], and presently, there are no practically useable technologies to remove inorganic ions such as phosphorus and nitrogen during sewage treatment using activated sludge methods. Thus, though various types of water purification systems have been developed for water and sewage plants [8], these technologies are often difficult to apply directly to aquatic environments due to cost and the need for special equipment. Eutrophication therefore remains a problem. 

Concurrently with improving sewage treatment technology, a low-cost and highly efficient method is still needed for sustainable water purification in aquatic environments. A treatment for environmental pollution using plants (phytoremediation) is a possible solution [9, 10]. Since phosphorus is an essential and often limiting nutritive substance for plants, plants actively absorb it from environments through the roots. Phytoremediation of aquatic systems has been attempted using water plants such as water hyacinth and *Phragmites*, as these plants absorb phosphorus relatively efficiently in comparison to terrestrial plants, and they also grow rapidly [11]. However, the high cost of collection and disposal of water plants (especially water hyacinth) presents difficulties in habitat management, and the impact of the plants on preexisting ecosystems hamper their wide application. In addition, the ability of these water plants to eliminate phosphorus in aquatic ecosystems is still inadequate as an even higher efficiency is needed for effective phytoremediation.

Inorganic phosphate (Pi) transporter is a key component in Pi absorption by plant roots. In *Arabidopsis thaliana*, 9 high-affinity transporters are known [12]. One of these, At*PHT1,* encodes a cell membrane-located Pi transporter with high affinity for Pi. It has been reported that overexpression of At*PHT1* in cultured cells of *Nicotiana* leads to an acceleration of Pi absorption and an increase in cell growth rate [13]. In contrast, when the same Pi transporter was overexpressed in *Hordeum vulgare*, an increase in absorption of Pi was not observed [14]. These two contradicting reports suggest that merely increasing the number of Pi transporters does not necessarily lead to enhanced Pi absorption.

Several Pi starvation-related genes have been identified in *A. thaliana* mutants [15]. One of the known control factors which function when plants enter a state of Pi starvation is the At*PHR1* gene. At*PHR1* gene encodes a transcription factor which activates the transcription of genes in response to states of Pi starvation [16]. Recently, it is reported that overexpression of At*PHR1 *in *A. thaliana* increases the Pi concentration in aerial plant parts [17].

In this study, we introduced the At*PHR1* gene into the garden plants *Torenia*, *Petunia,* and *Verbena*, in order to enhance Pi absorption. Small and large-scale hydroponic trials with transgenic torenia plants expressing the At*PHR1 *gene were performed. We demonstrate for the first time that over expression of the At*PHR1* gene results in enhanced Pi absorption rate in different plant species. The At*PHR1* transgenic plants can possibly facilitate effective phytoremediation in polluted aquatic environments. 

## 2. Materials and Methods

### 2.1. Plant Materials

Plants of *Torenia hybrida* cv. Summer Wave blue, *Petunia hybrida* cv. Surfinia purple mini, and *Verbena hybrida* cv. Temari scarlet (Suntory Flowers, Ltd.) were grown in soil and supplied with full nutrients every week in a green house or a growth chamber in controlled conditions (22–25°C, 12 hours light). 

### 2.2. Constructs for Expression in Plants and Plant Transformation

Molecular biology techniques were employed according to the methods described by Sambrook et al. [18], unless otherwise specified.

The At*PHR1* gene was amplified by PCR using primers PHRf (5′-ATGGAGGCTCGTCCAGTTCAT-3′) and PHRr (5′-TCAATTATCGATTTTGGGACGC-3′) and subcloned into the pCR2.1 vector using a TOPO-TA cloning kit (Life Technologies) according to the manufacturer's instructions. A fragment of the At*PHR1* gene was inserted into binary vector pBinPLUS [19] which contains an enhanced cauliflower mosaic virus 35S promoter [20] and a nopaline synthase (nos) terminator. This plasmid was named pSPB1898.

Transformation with transformation vector pSPB1898 was carried out as described previously for Torenia [21], Petunia [22], and Verbena [23] using *Agrobacterium tumefaciens *strain AGL0 [24].

RNAs were extracted from leaves of the obtained recombinant plants using the RNeasy Plant Mini Kit (Qiagen). Positive strains were selected by RT-PCR.

### 2.3. Method for Measuring Phosphorus Concentration

Phosphorus concentration was measured according to a modified method of Ames [25]. Leaves were weighed (approximately 100 mg per sample) and inserted into a 2 mL tube for crushing with zirconia beads (4 mm diameter), at −80°C. The frozen sample was taken to room temperature, and 500 *µ*L of 1% (v/v) acetic acid was added to each tube. The mixture was then shaken and crushed for 6 minutes using a TissueLyser (Qiagen). After crushing, the mixture was centrifuged at 15,000 rpm for 5 minutes using a desktop centrifuge to obtain 500 *µ*L of supernatant. This Pi extract was diluted with distilled water (from 10 to 100-fold dilution) to a final concentration of 800 *µ*L. To this solution, 160 *µ*L of measuring buffer (1.25 M sulfuric acid, 30 mM ascorbic acid, 0.405 mg/mL antimony potassium tartrate, and 24 mg/mL ammonium molybdate) was added, and the mixture was stirred well and left for 10 minutes. The absorbance was measured at 880 nm using a spectrophotometer BioSpec-mini (Shimadzu, Japan). The amount of phosphorus in 1 g of leaf was calculated from phosphorus concentration and weight of the sample. For calculations on a dry weight basis, samples were dried at 80°C for about 2 days.

An independent Student's *t*-test was used to compare differences between host and transgenic plants. All tests were two-sided, and *P* < 0.05 was considered statistically significant. Data are the mean ± SD from at least three different samples.

### 2.4. Hydroponic Experiment

Wild-type torenia or transgenic torenia was grown on a support made of polystyrene foam with holes to allow the root systems of the plants to grow into the hydroponic solution. Plants were floated on 5 liter of hydroponic solution (0.5 mM KNO_3_, 0.2 mM MgSO_4_, 0.2 mM Ca(NO_3_)_2_, 0.161 mM KPO_4_, 5 *µ*M Fe-EDTA, 7 *µ*M H_3_BO_3_, 1.4 *µ*M MnCl_2_, 0.05 *µ*M CuSO_4_, 0.1 mM ZnSo_4_, 0.02 *µ*M Na_2_MoO_4_, 1 *µ*M NaCl, and 0.001 *µ*M CoCl_2_). The initial phosphorus concentration in the hydroponic solution was 5 mg/L. Four plants were used in each support. The Pi concentration in the hydroponic solution was measured each day. Since the fluid volume of the hydroponic solution decreased due to transpiration and evaporation, on every fourth day, deionised water was added to the solution. For large container experiments, the same solution was used, but the volume of hydroponic solution was 400 liter, and 13 plants were used per container. The volume of each container was adjusted with deionised water on a weekly basis.

## 3. Results and Discussion

### 3.1. Overexpression of At*PHR1* Enhances Pi Accumulation and Absorption in Transgenic Plants

**Figure 1:**
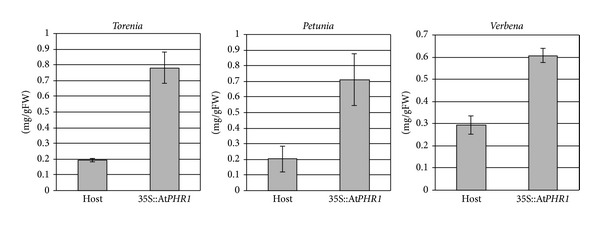
Phosphorus measurements of At*PHR1* transgenic plants. Phosphorus concentrations in the leaves of At*PHR1* transgenic plants of potted torenia, petunia, and verbena were measured. The longitudinal axis shows the phosphorus amounts per gram fresh weight (mg/gFW). Significant differences in means between host and transgenic plants were detected for all three species.

It has been shown in *A. thaliana* that over expression of At*PHR1* causes enhanced Pi accumulation in aerial parts [17]. To examine whether At*PHR1* is effective in otherplant species, we transformed torenia, petunia, and verbena with At*PHR1*. These plants were transformed with the plasmid pSPB1898, which contains the At*PHR1* gene under the control of the constitutive 35S promoter. We screened over 30 transgenic plants for each species for the presence of the transgene with RT-PCR and for leaf Pi concentration 4 weeks after potting up from tissue culture. Concentration of phosphorus per fresh leaf weight was then measured for selected lines. In each of the 3 plant species, phosphorus concentration in the leaves of the transgenic plants was 2 to 3-fold higher than that of control host plants (Figure 1). 

We examined other Pi starvation-related genes (At*PHT1;1*, At*PHT1;2*, At*IPS1,* and At*PHO1*) from *A. thaliana* by constitutively overexpressing them in transgenic torenia and petunia (data not shown). None of these transgenic plants showed enhanced Pi accumulation. This result is consistent with the observation that over-expression of the Pi transporter did not cause any change to Pi accumulation in *H. vulgare *[14]. Thus, we focused on At*PHR1* in the following experiments.

**Figure 2:**
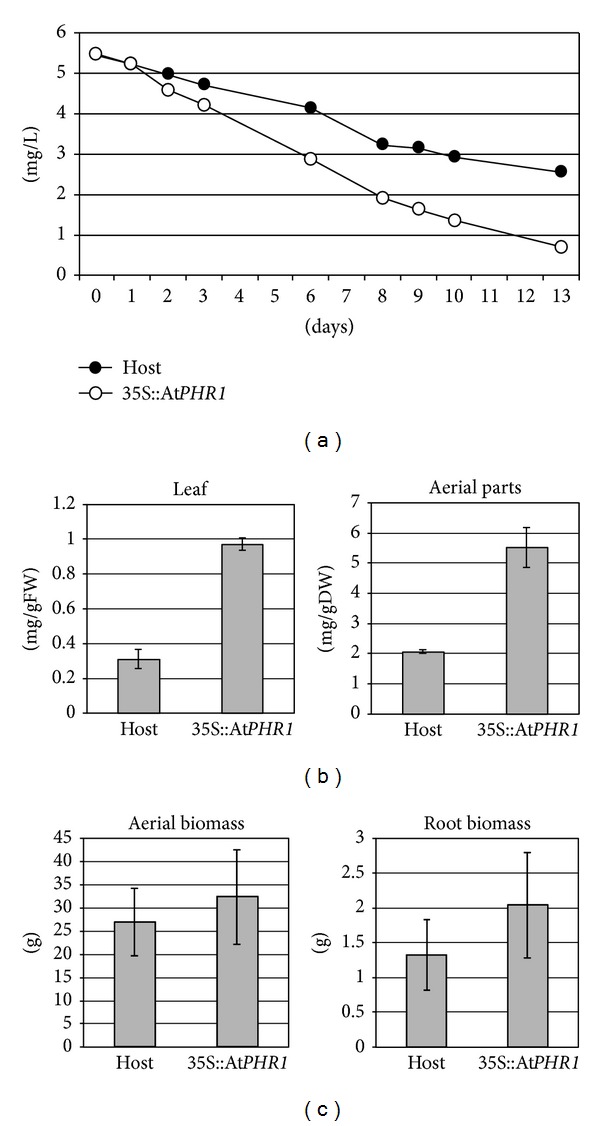
Pi accumulation and growth properties of At*PHR1* transgenic torenia. (a) Changes of Pi concentration in hydroponic solutions. The phosphorus concentration in a hydroponic solution in which host (filled circle) and At*PHR1* transgenic torenia (empty circle) were cultured was measured. The longitudinal axis shows the phosphorus concentration (mg/L), and the horizontal axis shows the number of days after exchange of the hydroponic solution. (b) Pi concentration in the leaves and aerial parts of hydroponically-cultivated torenia. The longitudinal axis shows the phosphorus concentration per gram fresh weight of samples (mg/gFW) (left) and the phosphorus concentration per gram dry weight of samples (mg/gDW) (right). There were significant differences in means between host and transgenic plants. (c) Comparison of growth rate. Weights of aerial parts and root parts of the torenia plants were measured at the end of hydroponic experiments. There was no statistically significant difference between transgenic and host.

To confirm that introduction of the At*PHR1* gene accelerates Pi absorption rates, we grew plants of a transgenic torenia line in a hydroponic system. Torenia was chosen as this plant grows luxuriantly and roots tolerate being submerged in water. The torenia plants were grown in 5 liters of hydroponic solution containing 5 mg/L phosphorus for 1 to 2 months in a green house or a growth chamber. The phosphorus concentration of the hydroponic solutions was measured daily. The superior transgenic line expressing At*PHR1* (35S::At*PHR1*) showed enhanced Pi absorption from the hydroponic solution (Figure 2(a)). Enhanced accumulation of Pi in the transgenic leaves was also confirmed by measurements of leaf phosphorus concentration (Figure 2(b)). The phosphorus concentration of the hydroponic solution in which 35S::At*PHR1* was grown decreased during the two weeks of the experiment. The Pi absorption rate observed for 35S::At*PHR1* was up to 0.091 mgP/day/plant in this experiment compared to 0.056 mgP/day/plant for the host (Figure 2(a)). This result suggests that the enhanced Pi accumulation observed in the potted At*PHR1* transgenic torenia plants is mainly due to enhanced Pi absorption rate. 

To see if the decrease of Pi concentration in the hydroponic solution was also reflected in an increase in Pi accumulation in the plant, Pi accumulation in the aerial parts of the plants was measured. Three plants each of the transgenic and the host torenia were hydroponically cultivated in the solution containing 5 mg/L phosphorus for about 2 months. The aerial parts of those plants were collected and dried on the phosphorus concentration measured (Figure 2(b)). The Pi concentration in the transgenic plants was approximately 2.5-fold that of the host. 

We weighed aerial and root parts of the tested plants after each hydroponic experiment. Even though slightly less weight was measured in the host, there was no statistically significant difference between the transgenic and host (Figure 2(c)). This suggests that excessively absorbed Pi is not used for plant growth but is accumulated and stored in the aerial part of the plants. As a result, overexpression of At*PHR1* does not retard plant growth. Since the transgenic plants did not show any morphological or reproductive abnormalities, over-expression of the At*PHR1* gene can enhance Pi accumulation with no negative effects on plant growth. 

### 3.2. Limitation of Pi Capacity

**Figure 3:**
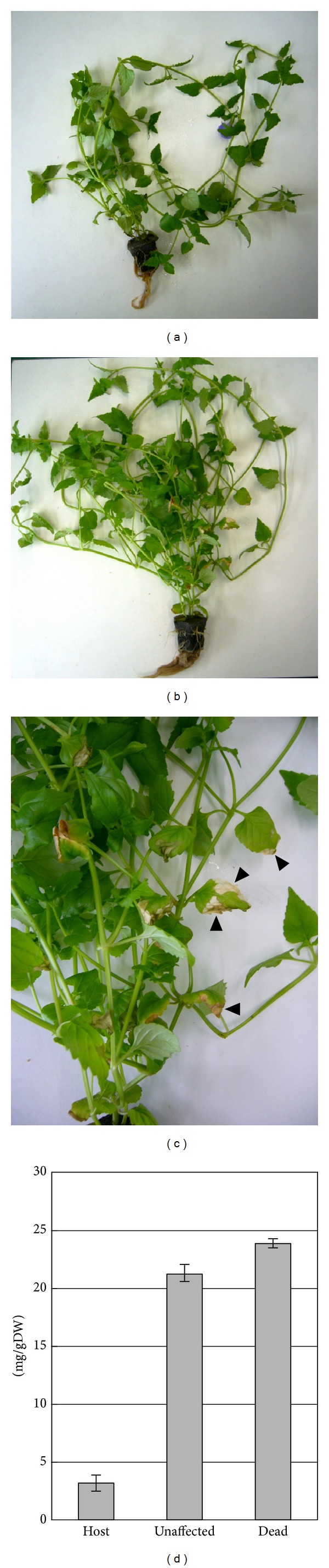
Dead tissue in At*PHR1* transgenic torenia. (a) Host plant at the end of hydroponic experiment. (b) At*PHR1* transgenic plant after 4 weeks of hydroponic experiment. (c) Magnified image of (b). Arrowheads indicate partially dead sections. (d) Phosphorus concentration in unaffected and dead areas from leaves of host and At*PHR1* transgenic plants. The longitudinal axis shows the phosphorus concentration per gram dry weight of sample (mg/gDW).

Sections of dead tissues in the leaves were often observed in transgenic torenia during the 4 weeks of the hydroponic experiments (Figures 3(a)–3(c)). We collected the dead sections and compared them to the unaffected areas of the leaves from the same plants. The harvested leaves were dried and then measured for phosphorus concentration. The phosphorus concentration in the dead sections was slightly higher than that of unaffected portions of leaves (Figure 3(d)). Since excess Pi may cause cell toxicity [26], the death may have been the result of exceeding a critical limit of Pi concentration in the torenia leaf cells. It thus appears that the critical limit of Pi accumulation level in At*PHR1* transgenic torenia is approximately 20 mg/gDW. One possible way to overcome the death of leaf tissues due to high Pi accumulation is to convert Pi to a nontoxic form of phosphorus that is phytic acid. Genetic modification could be used to achieve this, resulting in transgenic plants accumulating even more Pi than reported here. 

### 3.3. Large-Scale Hydroponic Experiment

**Figure 4:**
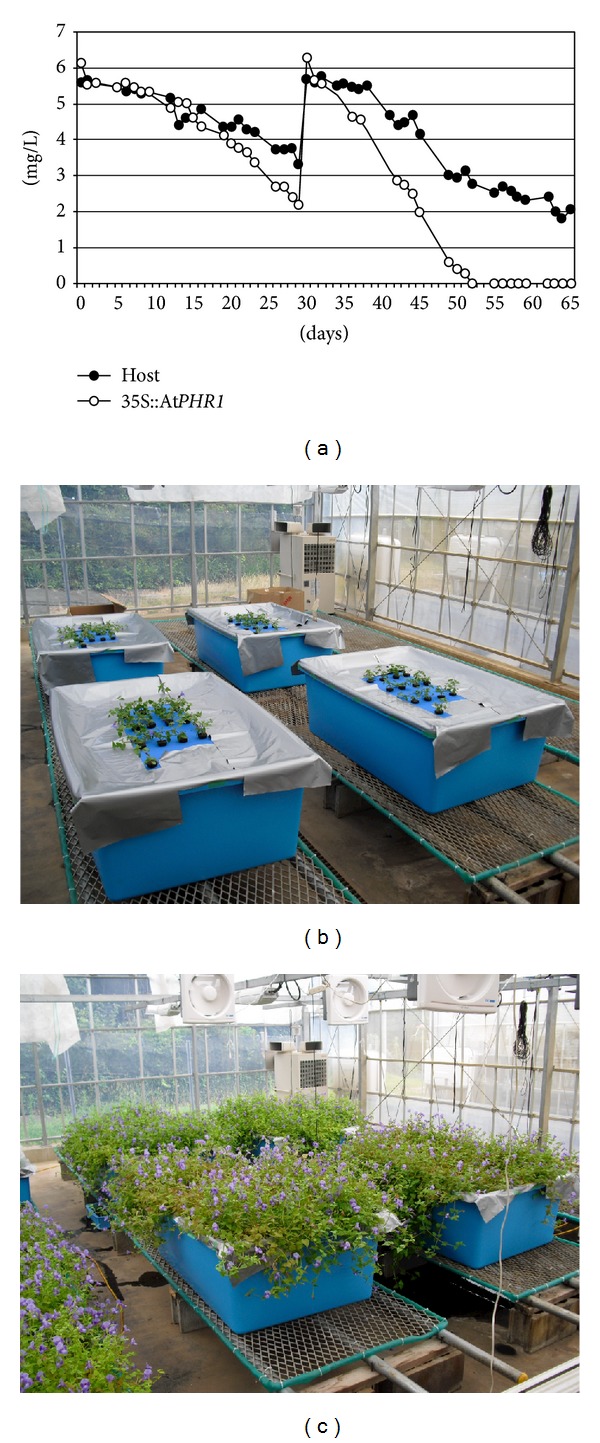
Large-scale hydroponic experiments. (a) Changes in Pi concentration in hydroponic solutions. The phosphorus concentration in a hydroponic solution in which host (filled circle) and At*PHR1* transgenic torenia (empty circle) were cultured was measured. The longitudinal axis shows the phosphorus concentration (mg/L), and the horizontal axis shows the number of days. Hydroponic solutions were fully exchanged 30 days after starting the experiment. (b) Large-scale experiment (0 day). (c) Large-scale experiment (65 days).

 To access the potential for phytoremediation using the transgenic torenia at a larger scale, we performed longer term hydroponic experiments. Thirteen torenia plants were put each into 400-liter tub and incubated for approximately 2 months (Figure 4). There was no significant difference in average biomass between transgenic and host plants after 65 days incubation (Figure 4 and Table 1). However, approximately 3-fold more Pi accumulation was seen in the transgenic plant when compared to the host. This confirmed that transgenic torenia shows the accelerated absorption as well as accumulation of Pi in the leaves when grown on a larger scale. From the daily calculation of Pi accumulation of the transgenic torenia plant, Pi accumulation rates were able to be compared to water hyacinth (Table 1). The At*PHR1* transgenic torenia showed an equivalent efficiency of Pi accumulation to that of water hyacinth [27, 28].

Overexpression of At*PHR1* gene might drive a Pi starvation response in the transgenic plants. As a result, excessive amounts of Pi accumulated in transgenic leaves. In *A. thaliana*, At*PHR1* gene is not transcriptionally regulated even under Pi starvation condition [17]. Since the key mechanism of the Pi starvation response is still debatable in *Arabidopsis thalinana* [17, 29], it is difficult to postulate why overexpression of At*PHR1* is effective for Pi uptake in other species. We have isolated orthologous Pi starvation-related genes (At*PHR1*, At*IPS1*, At*PHT1;1*, and At*PHO2*) in torenia and examined expression pattern of these genes (data not shown). We could not detect any differences between transgenic torenia and host plants. Overexpression of At*PHR1* may interfere with the proper posttranscriptional modification of the endogenous At*PHR1* counterpart, possibly through competitive inhibition.

Since phosphorus is expected to be exhausted as a natural resource within a hundred year [30], it is necessary to recover phosphorus from the environment, especially in polluted areas. Currently, over 90% of the produced phosphorus in the world is used as fertilizers. Therefore, it is most reasonable to recover phosphorus from fertilized soils and agricultural run-offs. Phytoremediation is a suitable method for such a recycling process, in addition to cleaning up phosphorus from the aquatic environment. One of the critical problems of phytoremediation is the cost of the disposal of the plant [31]. The plant used for phytoremediation was in many cases simply discarded without being used as a source of Pi. Ideally, plants containing high accumulation of Pi can be returned to soils of agricultural land without processing and can be directly used as fertilizer. However, at present, absorbing ability of the existing plants used for phytoremediation is not efficient enough to be used as Pi sources for agriculture in this way. In this study, the At*PHR1* transgenic plants accumulated a high level of Pi. Therefore, applications of At*PHR1* transgenic plants for phytoremediation of water could be cost-effective. Moreover, the Pi recycling ability of flowers and ornamental plants for gardening can be increased by means of At*PHR1* gene introduction, and thereby purifying water with plants having both ornamental beauty and high purification ability.

## 4. Conclusions

In this study, we prove the feasibility of using At*PHR1* as an enhancer of Pi uptake in transgenic plants. By introducing At*PHR1* to garden plants, amounts of Pi accumulation and absorption of Pi were increased to rates approximately 3-fold higher than host plant. There was no significant reduction in biomass or morphology of the transgenic plant expressing At*PHR1*. Taken together, these observations indicate that the At*PHR1* gene will be valuable for production of hyperaccumulator plants for the purification of waters polluted with Pi. In addition, an improved appearance of purification sites can be provided by using ornamental plants with many flowers, as shown in Figure 4(c).

## Figures and Tables

**Table 1:** Comparison of phosphate absorption performances. Phosphorus content, total biomass, and absorption rate after 65 days of the hydroponic experiment are indicated. Data are the mean ± SD from 13 plants. Values of water hyacinth were calculated from values listed in [27, 28].

	Phosphorus in leaf (mg/gFW)	Total biomass (g/plant)	Absorption rate (mg/plant/day)
Host	0.18 ± 0.11	396.34 ± 146.06	1.08
35S::At*PHR1 *	0.69 ± 0.20	382.95 ± 178.85	4.15
Water hyacinth	0.38		1.79
